# BDNF Polymorphism Predicts General Intelligence after Penetrating Traumatic Brain Injury

**DOI:** 10.1371/journal.pone.0027389

**Published:** 2011-11-08

**Authors:** Elham Rostami, Frank Krueger, Serguei Zoubak, Olga Dal Monte, Vanessa Raymont, Matteo Pardini, Colin A. Hodgkinson, David Goldman, Mårten Risling, Jordan Grafman

**Affiliations:** 1 Department of Neuroscience, Karolinska Institutet, Stockholm, Sweden; 2 Department of Molecular Neuroscience, George Mason University, Fairfax, Virginia, United States of America; 3 Department of Psychology, George Mason University, Fairfax, Virginia, United States of America; 4 Cognitive Neuroscience Section, National Institute of Neurological Disorders and Stroke, National Institutes of Health, Bethesda, Maryland, United States of America; 5 Laboratory of Neurogenetics, National Institute of Alcohol Abuse and Alcoholism, National Institutes of Health, Bethesda, Maryland, United States of America; 6 Center for Neuroscience and Regenerative Medicine at the Uniformed Services University of the Health Sciences, Bethesda, Maryland, United States of America; 7 Department of Neuroscience, Ophtalmology and Genetics, University of Genoa, Genoa, Italy; 8 Magnetic Resonance Research Centre on Nervous System Diseases, University of Genoa, Genoa, Italy; 9 Henry Jackson Foundation for Advancement in Military Medicine, Rockville, Maryland, United States of America; 10 Traumatic Brain Injury Research Laboratory, Kessler Foundation Research Center, West Orange, New Jersey, United States of America; 11 Johns Hopkins University, Department of Radiology, Baltimore, Maryland, United States of America; 12 Department of Radiology, Johns Hopkins University, Baltimore, Maryland, United States of America; 13 Department of Medicine, Imperial College, London, United Kingdom; Hangzhou Normal University, China

## Abstract

Neuronal plasticity is a fundamental factor in cognitive outcome following traumatic brain injury. Brain-derived neurotrophic factor (BDNF), a member of the neurotrophin family, plays an important role in this process. While there are many ways to measure cognitive outcome, general cognitive intelligence is a strong predictor of everyday decision-making, occupational attainment, social mobility and job performance. Thus it is an excellent measure of cognitive outcome following traumatic brain injury (TBI). Although the importance of the single-nucleotide polymorphisms polymorphism on cognitive function has been previously addressed, its role in recovery of general intelligence following TBI is unknown. We genotyped male Caucasian Vietnam combat veterans with focal penetrating TBI (pTBI) (n = 109) and non-head injured controls (n = 38) for 7 BDNF single-nucleotide polymorphisms. Subjects were administrated the Armed Forces Qualification Test (AFQT) at three different time periods: pre-injury on induction into the military, Phase II (10–15 years post-injury, and Phase III (30–35 years post-injury). Two single-nucleotide polymorphisms, rs7124442 and rs1519480, were significantly associated with post-injury recovery of general cognitive intelligence with the most pronounced effect at the Phase II time point, indicating lesion-induced plasticity. The genotypes accounted for 5% of the variance of the AFQT scores, independently of other significant predictors such as pre-injury intelligence and percentage of brain volume loss. These data indicate that genetic variations in BDNF play a significant role in lesion-induced recovery following pTBI. Identifying the underlying mechanism of this brain-derived neurotrophic factor effect could provide insight into an important aspect of post-traumatic cognitive recovery.

## Introduction

Traumatic brain injury (TBI) is a major public health concern in industrialized countries. It is estimated that 1.7 million people sustain a TBI annually in the United States. Young children, adolescents and the elderly, predominantly male, suffer the highest rates of TBI [1]. The treatment and improvement of outcome in TBI subjects still remains a challenge [2]. Previous studies have identified pre-injury general intelligence as the best predictor of cognitive outcome after TBI [3], [4], [5]. Further, general intelligence is a strong predictor of occupational attainment, social mobility and job performance as well as everyday decision making [6], [7] and thus is an excellent measure of outcome and neuronal plasticity in TBI subjects.

Brain-derived neurotrophic factor (BDNF), the most abundant neurotrophin in the brain [8], [9], plays an important role in the survival, differentiation, synaptic plasticity and outgrowth of peripheral and central neurons throughout adulthood [10], [11], [12]. The BDNF gene is located in the short arm of chromosome 11. Several hundred polymorphisms in the BDNF gene have been reported so far and two of them are shown to impact the expression level of BDNF. The first one is BDNF-LCPR (BDNF-linked complex polymorphic region), a 5′-UTR located microsatellite polymorphism, that is associated with an elevated risk of bipolar disorder and a lower transcription level of BDNF [13]. The second BDNF polymorphism is the SNP rs6265, which produces a missense mutation Val66Met (196G/A) and affects the regulated secretion and neuroplastic effect of mature BDNF [14].

Several studies have shown an association between the BDNF polymorphism and episodic memory [14] and hippocampal volumes [15], [16], [17], [18]. An association between a BDNF polymorphism and different neuropathological conditions such as Alzheimer's disease [19], obsessive-compulsive disorder [20], eating disorder [21] and bipolar disorder [22], [23] has also been documented, as well as an association with executive functions in TBI subjects [24]. In addition, studies in experimental TBI have shown increased expression of BDNF in hippocampus following focal injury [25]. However, no association has yet been shown between the BDNF polymorphism and general cognitive intelligence in a healthy population [5], [14], [26]. Due to its strong influence on brain plasticity we hypothesized that the BDNF polymorphism would affect the recovery of general cognitive intelligence after penetrating TBI (pTBI).

Military personnel represent an ideal population when studying changes in cognitive functions after pTBI, since pre- and post-injury data are usually available in the form of the Army Force Qualification Test (AFQT) on which performance is associated highly with scores on the Wechsler Adult Intelligence Scale (WAIS) [3]. Thus, we investigated the association of 7 SNPs within the BDNF gene region with recovery of general cognitive intelligence after focal pTBI in male Vietnam combat veterans 10–15 years (Phase II) and 30–35 years after injury (Phase III).

## Materials and Methods

### Subjects

Veterans were drawn from Phase III of the W.F. Caveness Vietnam Head Injury Study (VHIS) registry. The VHIS is a prospective, long-term follow-up study of veterans with mostly focal pTBIs, which has stretched over more than 3 decades [5], [27] The VHIS registry was collected during the Vietnam conflict by William Caveness at the National Institutes of Health (NIH). Simple registry forms outlining demographic, injury and initial outcome data were completed by military physicians in Vietnam on head injured soldiers who had survived the first week after a severe head-injury including information about “type of penetrating head injury” and “classification of loss of consciousness”. About 2,000 subjects were entered in the registry between 1967 and 1970. Phase I (PI) of the VHIS was a medical records review some 5 years post-injury using the military, VA medical and personnel records of 1221 of these men, for whom adequate field, hospital, rehabilitation and follow-up records were available.

Phase II (PII) was a collaborative project of the three Military Services; the Department of Veterans Affairs, the National Institutes of Health and the American Red Cross. It consisted of a comprehensive, multidisciplinary inpatient evaluation at Walter Reed Army Medical Center. Approximately 520 head injured subjects from the original registry could be identified from VA records, so these and 85 matched normal volunteers (recruited through veteran publications) were evaluated between 1981 and 1984, some 12–15 years post-injury.

At Phase III (PIII), of the 520 subjects who were assessed in P2, 484 were still alive and 182 attended P3 of the study. In addition, 17 subjects identified in PI who did not attend PII were assessed. Of 10 the original 80 control subjects without head injuries recruited in PII, 32 attended P3 and a further 23 were recruited for PIII through advertisements in veteran publications. Therefore, a total of 199 subjects with head injuries attended PIII. No significant differences in age were observed between PIII attendees and non-attendees, in both the head-injured and control groups. However, subjects (both head-injured and healthy controls) that attended PIII did have more years of education (t = 3.06, P = <0.002), and higher AFQT scores (pre-injury: t = 4.85, P<0.001, PII: t = 6.15, P<0.001) than PIII non-attendees. Since those subjects attending PIII had a higher level of pre-injury intelligence than those attending PII, as well as more years of education, it is possible that those studied at PIII differed in other ways from PIII non-attendees, which may have affected the longitudinal results we report in this paper.

A further reduction of sample size in this study is explained by several reasons: First, out of these 199 only 168 consented to genotyping. Second, from the remaining subjects those who did not complete all three phases of the study were excluded from the analyses (n = 33). Third, as a majority of studied subjects were Caucasian in ethnicity, those subjects who had Caucasian ethnicity AIM scores <0.5 were also excluded (n = 25). Finally, one subject had to be excluded as an outlier due to his massive brain volume loss. The final studied samples included male Caucasian combat veterans with focal pTBIs (n = 109) and non-head-injured normal control subjects who also served in Vietnam (n = 38). Importantly, there were no significant differences in AFQT scores at PIII or educational level attained in the group of 109 we studied compared to the 90 excluded head-injured subjects from PIII (F(1,193) = 0.10, P = 0.919). The type of pTBI injury was classified by neurosurgeons at the time of injury into the following categories: Fragment (69.1%), Gunshot (21.3%), Unclassified (1.5%) and Closed Head injury (8.1%). Further, loss of consciousness (LOC) was classified as following: No (42.6%) Yes, Momentary (17.6%), 1–15 min (14%), 15 min – 1 day (11.8%), >1 day (11%), unknown (1.5%). The finals groups were matched with respect to age, level of education, and pre-injury intelligence (**Table 1**). All participants gave their written informed consent, which was approved by the Institutional Review Board at the National Naval Medical Center and the National Institute of Neurological Disorders and Stroke, Bethesda, MD, USA.

**Table 1:** Demographics variables and AFQT scores for the injured and control groups.

Variable	Injured Group (n = 109)	Control Group (n = 38)	Statistics	P-values
Education (years)	15.1±2.2	15.6±2.4	*t* = 1.07	0.285
Age (years)	58.8±2.8	58.2±3.1	*t* = 1.28	0.203
Pre-injury AFQT(percentile)	67.8±20.5	74.0±17.2	*t* = 1.51	0.132

### Neuropsychological Testing

Subjects were admitted at the National Naval Medical Center in Bethesda, MD, over a 5–7 day period and underwent a wide variety of neuropsychological testing. The tests were designed to measure cognitive abilities such as memory, language, social cognition, and executive functioning. In this study, we focused on the AFQT (AFQT-7A, Department of Defense, 1960), which is a standardized multiple choice test of cognitive aptitude measuring verbal ability, visual-spatial organization, arithmetic and functional associations via multiple choice questions. The total score range is 0 to 100 and the subtest scores range from 0 to 25. AFQT scores are reported as percentiles (1 to 99) and correlate highly with WAIS scores [3]. It was the only pre-injury cognitive assessment available in our sample and was also used in PII and PIII.

To determine the specific effect of BDNF genotype on the recovery of general cognitive intelligence, two additional cognitive control tasks were used in this study: First, the mini-mental state examination test (MMSE) from PIII was used, which is a well-validated standard test for cognitive impairment in adults, where scores <24 indicates cognitive impairment [28]. The purpose of its inclusion was to separate out issues of exacerbated cognitive decline from the onset of dementia [5]. Second, the delayed score of the logical memory subtest of the Wechsler Memory Scale, version III (WMS-III) was used to assess episodic memory, which reflects the amount of information from stories that a subject can recall after a 30 min delay [29].

### Computed Tomography (CT) Acquisition and Analysis

The axial CT scans were acquired without contrast in helical mode on a GE Electric Medical Systems Light Speed Plus CT scanner at the Bethesda Naval Hospital. Structural neuroimaging data was reconstructed with an in-plane voxel size of 0.4×0.4 mm, an overlapping slice thickness of 2.5 mm and a 1 mm slice interval. The lesion location and volume were determined from CT images using the interactive Analysis of Brain Lesions (ABLe) software implemented in MEDx v3.44 (Medical Numerics) [30], [31]. The analysis was performed on CT images from Phase III. Lesion volume was calculated by manually tracing the lesion in all relevant slices of the CT image in native space, and then summing the trace areas and multiplying by slice thickness. Manual tracing was performed by a trained psychiatrist (V.R.) with clinical experience of reading CT scans. The lesion tracing was then reviewed by an observer that was blind to the results of the clinical evaluation and neuropsychological testing (J.G.) enabling a consensus decision to be reached regarding the limits of each lesion. The CT image of each individual's brain was normalized to a CT template brain image in Montreal Neurological Institute (MNI) space. The spatial normalization was performed with the AIR algorithm [32], using a 12-parameter affine fit. Note that both the patient's brain and the MNI template's brain are first skull-stripped in order to maximize the efficacy of the AIR registration from native space to MNI space. In addition, voxels inside the traced lesion were not included in the spatial normalization procedure. Afterwards, the percentage of Automated anatomical labeling (AAL) structures that were intersected by the lesion was determined by analyzing the overlap of the spatially normalized lesion image with the AAL atlas [33].

### Genotyping and Haplotype Analysis

We used an addiction array designed by Hodgkinson et al [34]. The array is built on the Illumina GoldenGate platform and allows for simultaneous genotyping of 1350 SNPs including 7 BDNF SNPs (**Table 2**). The candidate genes for the array were selected on the basis of their roles in the drug addictions and the related phenotypes of anxiety and depression. These are the genes important in signaling networks, stress/endocrine genes, and key neurotransmitter systems including dopamine, serotonin, glutamate, GABA and acetylcholine. As all these functional domains are involved in the majority of brain functions, the addiction array represents very convenient tool for our study. For each gene (including BDNF) array contains SNPs that tag common haplotypes. In addition to 130 addiction-related genes, array includes a panel of 186 ancestry information markers (AIMs) that allows for determining subject's ethnicity background. Each marker represents a SNP with known frequencies of occurrence in different ethnical groups. The AIM panel covers seven major populations: African, European, Middle Eastern, Asian, Far East, Oceania and Native American. Following genotyping, the population assignment was performed for each individual according to obtained AIM scores. Only Caucasians with a “European” AIM score >0.5 were included in this study. Genotyping was performed according to the Illumina protocol on 96 well-format Sentrix arrays. The completion rate of array assay was >99%. The error rate of the assay was determined by replicate genotyping, and was <0.5%. Genotype frequencies were tested for the Hardy-Weinberg equilibrium (HWE) applying Fisher's exact tests. Haplotype analysis was performed using a Bayesian approach implemented with PHASE [35]. Haploview 4.2 (Broad Institute, USA,) was used to produce linkage disequilibrium (LD) matrices. Haplotype blocks were constructed by pairing the SNPs with the LD's greater than 0.85, as described by Gabriel et al [36]. We also investigated the effect of the presence of the ApoE e4 allele and COMT Val158Met (rs4680) on recovery of general intelligence to determine the relative specificity of any BDNF effect.

**Table 2:** Studied BDNF SNPs: chromosome position, location, minor allele frequencies and genotyping quality control values are present.

SNP	Chr. Pos^1^	Location	Ref MAF^2^	MAF	HW_Pval	PredHet^3^	ObsHet^4^
rs1519480	27632288	3′-flank	0.33	0.31	0.405	0.429	0.463
rs7124442	27633617	3′-UTR	0.33	0.31	0.405	0.429	0.463
rs6265	27636492	missense Val66Met	0.20	0.21	0.991	0.327	0.331
rs7934165	27688559	intron	0.43	0.45	0.751	0.495	0.514
rs11030121	27692783	intron	0.35	0.31	0.964	0.428	0.437
rs12273363	27701435	5′-flank	0.18	0.17	0.0185		
rs908867	27702340	5′-flank	0.12	0.11	0.808	0.189	0.200

1 Positions on chromosome 11 correspond to dbSNP build 126B and human genome build 36.3.

2 Reference minor allele frequencies are for Caucasian population (CEU, HapMap Release 28, Phase II+III, August 2010, NCBI B36 Assembly).

3 Predicted and

^4^observed heterozygote percentages.

5 SNP rs12273363 was excluded from the analysis because of lack of consistency with Hardy-Weinberg equilibrium test.

### Statistical Analysis

Behavioral data analysis was carried out using SPSS 15.0 with an alpha level set to p<0.05 (two-tailed). Multiple comparisons with Bonferroni correction were included in all analyzes. The relationship between variations in the BDNF genotype and the recovery of general cognitive intelligence was analyzed in several ways:

First, the demographic variables between the injured and controls groups were compared to ensure that the groups were matched with respect to age, education, and pre-injury AFQT using one-way analysis of variances (ANOVAs) with Group (injured, control) as a between-subjects factor.

Second, the AQFT percentile score of the injured group was normalized (z-transformation) in comparison to the performances of the control group. For each of the 7 SNPs of the BDNF gene, a mixed 3×3 analysis of variance (ANOVA) on AQFT z-scores was performed with Time (pre-injury, PII, PIII) as a within-subjects factor and Genotype (TT, CT, CC) as a between-subjects factor. In planned follow-up analyses, the AFQT z- scores among the different allele carriers in each SNP were compared using between-subjects t-tests. In addition, effect sizes (Cohen's d) that represent the observed difference in the AFQT performance between genotype groups were calculated (d = 0.2 indicates a small effect size, d = 0.5 a medium effect size and d = 0.8 a large effect size) [37].

Third, the specificity of the BDNF genotype effect on the recovery of general cognitive intelligence was determined. Since the BDNF polymorphism has been shown to modulate episodic memory and hippocampal function (Egan et al., 2003; Dempster et al., 2005), episodic memory scores were compared among the different allele carriers in the injured and normal control groups applying a 2×3 ANOVA with Group (injured, control) and Genotype (TT, CT, CC) as between-subjects factors. In planned follow-up analyses, the episodic memory scores among the different allele carriers in each group were compared using between-subjects t tests. Subjects within the genotype groups did not differ in age, education, lesion size, pre-injury AFQT.

Fourth, the relative contribution of the BDNF genotype on the recovery of general intelligence was estimated for PII and PIII. A stepwise multiple linear regression analysis was applied including the AFQT z-score as the dependent variable and BDNF genotype, pre-injury intelligence, age, education, degree of atrophy, percentage of total brain volume loss and brain volume loss within each hemisphere as independent variables. This analysis allowed for an estimation of the relative contribution of each predictor to general intelligence. At the same time, it controls for potential confounding factors that may influence general intelligence.

Fifth, the influence of the ApoE e4 allele or COMT Val158Met genotype on the recovery of general intelligence was determined applying a mixed 3×3 analysis of variance (ANOVA) on AQFT z-scores with Time (pre-injury, PII, PIII) as a within-subjects factor and Genotype as a between-subjects factor.

Finally, we performed a haplotype analysis to increase the chances of capturing gene-disease association by applying an ANOVA on AQFT z-scores with Time (pre-injury, PII, PIII) as a within-subjects factor and haplotypes (111222, 112122, 222211, 222212) as a between-subjects factor. The ANOVA was done for 2 haplotype blocks: block 1 included rs1519480, rs7124442, and rs6265, whereas block 2 included rs7934165, rs11030121, and rs908867.

## Results

First, in terms of differences in demographic variables the one-way ANOVAs showed no significant differences between the injured and control groups regarding age, education, and pre-injury AFQT (**Table 1**).

**Figure 1:**
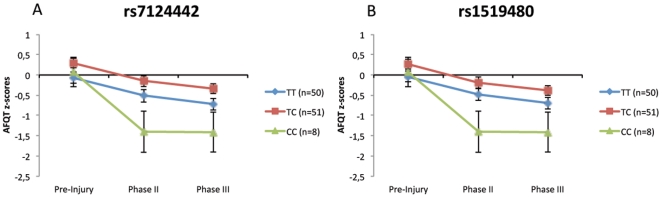
Association analysis of significant SNPs. The z-transformed (normalized) AFQT-scores of pre-injury, phase II and III for SNP are illustrated for the significant SNPs, rs7124442 (A) and SNP rs1519480 (B). Note that zero-line represents our normal control.

Second, the association between BDNF genotype and general cognitive intelligence at each evaluation Phase and for each of the 7 BDNF SNPs was evaluated. The mixed 3×3 ANOVAs on AQFT z-scores with Time (pre-injury, PII, PIII) and Genotype (TT, CT, CC) revealed a significant main effect of Time (*P*<0.001) but no significant main effect of Genotype for all SNPs (**Table 3**). The interaction effect for Time × Genotype was only significant for SNPs rs7124442 and rs1519480. For both SNPs, no differences were found regarding type of injury (rs1519480: *F*(2,108) = 0.105, *P* = 0.900; rs7124442: *F*(2,108) = 0.228, *P* = 0.797) and loss of consciousness (rs1519480: *F*(2,106) = 1,101, *P* = 0.336; rs7124442: *F*(2,106) = 1,043, *P* = 0.356). **Figure 1** illustrates the changes in AFQT z-scores across time (pre-injury, PII, PIII) among carriers of different genotypes of those SNPs rs7124442 and rs1519480. The genotype groups that performed best were CT (*n = 51*), in both SNP rs7124442 and rs1519480, and the worst groups were genotype CC (*n = 8*). For SNPs rs7124442 and rs1519480, the ANOVAs analysis showed only a significant decline of general cognitive intelligence from pre-injury to PII but not from PII to PIII (**Table 4**), indicating that those SNPs are more prominent during the early post-injury period, For both SNPs at PII, follow up t-tests revealed only significant differences between the CC and CT genotypes (**Table 5**).

**Table 3:** Association analysis of AFQT scores and SNPs.

SNP ID	Genotype	Injured Group	Time df(2,212)	Time × Genotype df(4,412)	Genotype df(2,106)
	TT	50			
rs1519480	CT	51	*F* = 44.2 *P*<0.001*	*F* = 3.5, *P* = 0.008*	*F* = 3.5, *P* = 0.033
	CC	8			
	TT	50			
rs7124442	CT	51	*F* = 44.2, *P*<0.001*	*F* = 3.6, *P* = 0.008*	*F* = 4.2, *P* = 0.017
	CC	8			
	AA	5			
rs6265	AG	33	*F* = 8.1, *P*<0.001*	*F* = 1.1, *P* = 0.356	*F* = 2.1, *P* = 0.125
	GG	71			
	AA	24			
rs7934165	AG	55	*F* = 44.0, *P*<0.001*	*F* = 0.7, *P* = 0.583	*F* = 3.1, *P* = 0.052
	GG	30			
	TT	10			
rs11030121	CT	46	*F* = 34.4, *P*<0.001*	*F* = 1.2, *P* = 0.325	*F* = 1.9, *P* = 0.155
	CC	53			
	TT	77			
rs12273363	CT	27	*F* = 21.9, *P*<0.001*	*F* = 0.72, *P* = 0.581	*F* = 0.1, *P* = 0.954
	CC	5			

The z-transformed (normalized) AFQT-scores were used to evaluate the association between BDNF genotypes and general intelligence at pre-injury, early- recovery and late- recovery. All 7 SNPs were analyzed using three-way ANOVA. Importantly, SNP rs908867 was excluded from the analysis because of low number of minor allele carriers. SNP rs12273363 was excluded from further analysis because of lack of consistency with Hardy-Weinberg equilibrium test.

*Effect survives Bonferroni correction (P = 0.05/6 ≈ 0.0083).

**Table 4:** The SNPs that showed to have a significant association were further analyzed to see the significant change from pre-injury to later and Phase II to Phase III.

SNP ID	Phase	Time df(1,106)	Time × Genotype Df(2,106)
rs7124442	Pre-injury to PII	*F* = 66.7, *P*<0.001*	*F* = 5.3, *P* = 0.006*
	PII to PIII	*F* = 2.3, *P* = 0.130	*F* = 0.3, *P* = 0.739
rs1519480	Pre-injury to PII	*F* = 66.7, *P*<0.001*	*F = *5.3, *P* = 0.006*
	PII to PIII	*F* = 2.3, *P* = 0.130	*F* = 0.3, *P* = 0.734

*Effect survives Bonferroni correction (P = 0.05/6 ≈ 0.0083).

**Table 5:** Results of t-test performed on the genotypes in the 2 significantly associated SNPs, comparing AFQT scores at each time point.

SNP ID	Genotype	Pre-injury	Phase II	Phase III
	TT vs CT (df = 99)	*t* = −1.95, P = 0.053; d = 0.39	*t* = 1.76, P = 0.081; d = 0.35	*t* = −1.95, P = 0.054; d = 1.12
rs7124442	CC vs CT (df = 56)	*t* = 0.68, P = 0.495; d = 0.76	*t* = −3.24, P = 0.002*; d = 0.70	*t* = −2.99, P = 0.004; d = 0.58
	CC vs TT (df = 57)	*t* = 0.35, P = 0.727; d = 0.17	*t* = −2.03, P = 0.047; d = 0.38	*t* = −1.68, P = 0.097; d = 0.26
	TT vs CT (df = 99)	*t* = −1.61, P = 0.112; d = 0.32	*t* = −1.43, P = 0.156; d = 0.28	*t* = −1.63, P = 0.105; d = 0.32
rs1519480	CC vs CT (df = 56)	*t* = −0.56,P = 0.573; d = 0.03	*t* = −3.08, P = 0.003*; d = 0.66	*t* = −2.86, P = 0.006; d = 0.63
	CC vs TT (df = 57)	*t* = 0.28, P = 0.780; d = 0.27	*t* = −2.11, P = 0.039; d = 0.40	*t* = −1.75, P = 0.085; d = 0.27

*Effect survives Bonferroni correction (P = 0.05/3 ≈ 0.017).

Third, the specificity of the BDNF genotype effect on the recovery of general cognitive intelligence was determined and no significant differences were observed for both SNPs (rs7124442, rs1519480) regarding demographic variables, volume loss, degree of atrophy, episodic memory and MMSE (SNP rs7124442: **Table 6**, SNP rs1519480: **Table 7**).

**Table 6:** Illustrates comparisons of demographics variables between the different genotypes of SNP rs7124442.

Variable	Diplotypes	Injured Group (n = 109) Mean±SD	F(2,108)	P-value
	TT (n = 51)	15.0±2.3		
Education (Years)	CT(n = 50)	14.8±1.2	0.12	0.882
	CC (n = 8)	14.9±3.7		
	TT (n = 51)	58.1±3.1		
Age (Years)	CT(n = 50)	57.4±2.2	0.65	0.522
	CC (n = 8)	58.4±3.6		
	TT (n = 51)	33.4±31.5		
Total Volume Loss	CT(n = 50)	34.6±34.3	0.42	0.656
	CC (n = 8)	45.3±32.2		
	TT (n = 51)	14.1±23.8		
Left Volume Loss	CT(n = 50)	17.7±24.2	1.05	0.352
	CC (n = 8)	27.6±27.1		
	TT (n = 51)	18,3±23.9		
Right Volume Loss	CT(n = 50)	16.0±23.6	0.08	0.915
	CC (n = 8)	17.7±25.3		
	TT (n = 51)	2.4±2.2		
Percent volume loss	CT(n = 50)	2.5±2.6	0.53	0.590
	CC (n = 8)	3.5±2.5		
	TT (n = 51)	63.7±22.4		
PRE INJ AFQT	CT(n = 50)	71.4±18.7	1.94	0.148
	CC (n = 8)	70.6±22.8		
	TT (n = 51)	28.7±1.8		
MMSE: Total Score	CT(n = 50)	28.1±1.3	0.51	0.598
	CC (n = 8)	28.4±1.0		
	TT (n = 51)	10.9±2.9		
Episodic memory	CT(n = 50)	10.3±3.3	0.55	0.576
	CC (n = 8)	11.1±3.1		

**Table 7:** Illustrates comparisons of demographics variables between the different genotypes of SNPrs1519480.

Variable	Diplotypes	Injured Group (n = 109) Mean±SD	F(2,108)	P-value
	TT (n = 51)	15,5±2.4		
Education (Years)	CT(n = 50)	14,8±1.4	0.16	0.847
	CC (n = 8)	14,0±3.3		
	TT (n = 51)	57,5±3.5		
Age (Years)	CT(n = 50)	57,4±2.2	0.52	0.594
	CC (n = 8)	58.1±3.1		
	TT (n = 51)	34,1±31.4		
Total Volume Loss	CT(n = 50)	34.3±34.4	0.40	0.666
	CC (n = 8)	45,6±32.9		
	TT (n = 51)	15.1±23.4		
Left Volume Loss	CT(n = 50)	17,8±24.9	0.97	0.381
	CC (n = 8)	27.3±27.3		
	TT (n = 51)	19,0±23.4		
Right Volume Loss	CT(n = 50)	16.4±23.5	0.15	0.857
	CC (n = 8)	17,3±25.4		
	TT (n = 51)	2.5±2.3		
Percent volume loss	CT(n = 50)	2.5±2.6	0.51	0.602
	CC (n = 8)	2,4±2.2		
	TT (n = 51)	64.5±21.8		
PRE INJ AFQT	CT(n = 50)	70,7±18.9	1.33	0.268
	CC (n = 8)	70.3±22.3		
	TT (n = 51)	28,5±1.9		
MMSE: Total Score	CT(n = 50)	28.1±1.2	0.54	0.584
	CC (n = 8)	28,7±1.0		
	TT (n = 51)	10.9±2.9		
Episodic memory	CT(n = 50)	10.2±3.3	0.80	0.452
	CC (n = 8)	11.1±3.1		

Fourth, the relative contribution of the significant SNPs to the recovery of general cognitive intelligence at PII and PIII was investigated using stepwise multiple linear regression analyzes. For phase II, variance in cognitive intelligence was explained by pre-injury intelligence (47.0%, P<0.001, Beta = 0.68), to SNP rs7124442 and genotype CC (4.9%, P<0.001, Beta = −0.221), percentage of total brain volume loss (2.1%, P<0.041, Beta = −0.152), and education (1.5%, P<0.018, Beta = 0.130). For phase III, variance in cognitive intelligence was explained by pre-injury intelligence (41.8%, P = <0.001, *R^2^* = 0.414, Beta = 0.647), percentage of total brain volume loss (6.4%, P<0.001, Beta = −0.253), and SNP rs7124442 and genotype CC (2.4%, P<0.018, Beta = −0.160). No significant associations were found for age, education, atrophy ratings or right or left hemisphere brain volume loss for both time-points.

Fifth, we determined whether the presence of the ApoE e4 allele or COMT Val158Met genotype has an effect on the recovery of general cognitive intelligence. This genetic analysis for ApoE and COMT was available for 94 of the subjects. In the injured group, 22 subjects showed presence of the ApoEe4 allele. The ANOVA revealed that there was a main effect on Time (*F*(2,182) = 25.2 *P*<0.001) but no main effect for Genotype (*F*(1,91) = 0.95, *P*<0.332) and no interaction effect for Time × Genotype (*F*(2,182) = 1.11, P<0.328). The influence for the COMT polymorphism (Val/Val = 23, Val/Met = 38, Met/Met = 33) was similar: significant main effect for Time (*F*(2,182) = 34.4, *P*<0.001) but no significant main effect for Genotype (*F*(4,182) = 2.5, *P*<0.086) and not significant interaction effect for Time × Genotype (*F*(2,91) = 1.4, *P*<0.247).

**Figure 2:**
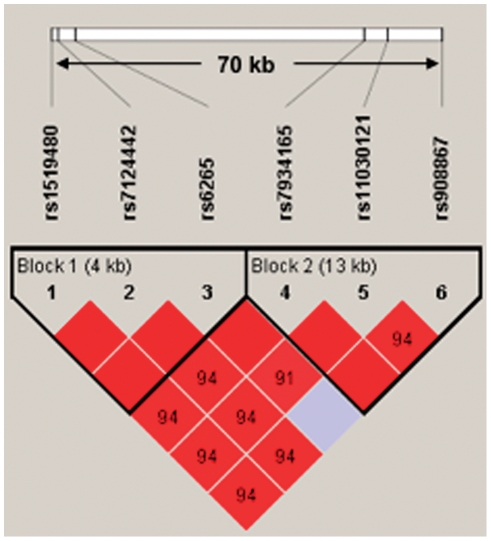
BDNF haplotype block structure. BDNF haplotype block structure. The numbers within the diamonds are D' values representing linkage disequilibrium between SNP pairs. Only values of D'<98 are present so the solid red diamonds represent absolute LD (D' = 1) and the grey diamonds represent high probability or evidence of historical recombination. Two short sub-blocks of 4 kb and 13 kb long were determined with use of Haploview software and used for detailed analysis. The blocks are defined using the pairs of SNPs with an LD >0.85. Note that BDNF gene is located on minus DNA strand.

**Figure 3:**
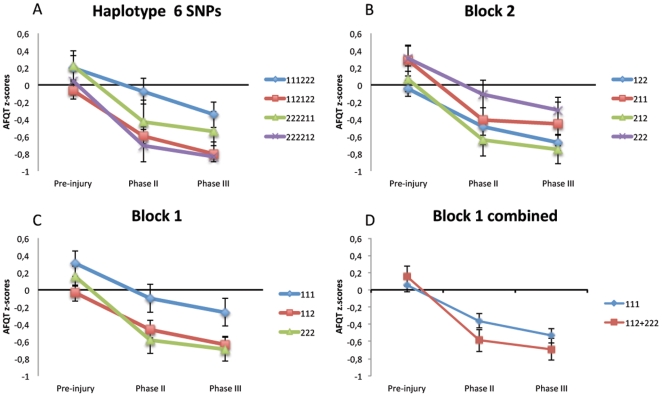
Haplotype association analysis. Haplotype association analysis illustrating z-Normalized AFQT scores among different genotype carriers pre-injury, Phase II and Phase III. Haplotype 6 SNPs (A) include all the SNPs in LD. Block 2 (B) includes SNPs rs7934165, rs11030121 and rs908867. Block 1 (C) includes SNPs rs1519480, rs7124442 and rs6265. In addition we analyzed the combination of haplotype 112 and 222 from block 1 and compared this to group 111 (D).

Finally, a haplotype analysis was carried out, since the haplotype association analysis of polymorphisms in strong LD can potentially be more suitable for identifying gene–disease associations than single locus tests. The BDNF haplotype block structure is presented in **Figure 2**. Note that the SNP rs12273363 was excluded from the analysis due to a lack of consistency with the Hardy-Weinberg equilibrium test. Four haplotypes with frequencies >0.09 were used for the analyses (**Table 8**). No significant associations were found for these haplotypes (data not shown). We further divided the main haplotype block into two short sub-blocks of 4 kb and 13 kb long. By Haploview software the blocks were defined using the pairs of SNPs with an LD >0.85. The presence of a recombination region between SNPs 3 and 4 suggested 2 blocks. Block 1 included rs1519480, rs7124442 and rs6265 and Block 2 consisted of rs7934165, rs11030121 and rs908867. Haplotype frequencies for sub-blocks are present in **Table 8**. Our ANOVA analysis showed a significant association for haplotype block 1 (combined) (P<0.017). A summary of the haplotype analysis can be found in **Table 9** and **Figure 3** illustrates the decline of AFQT z-scores over time for different haplotypes.

**Figure 4:**
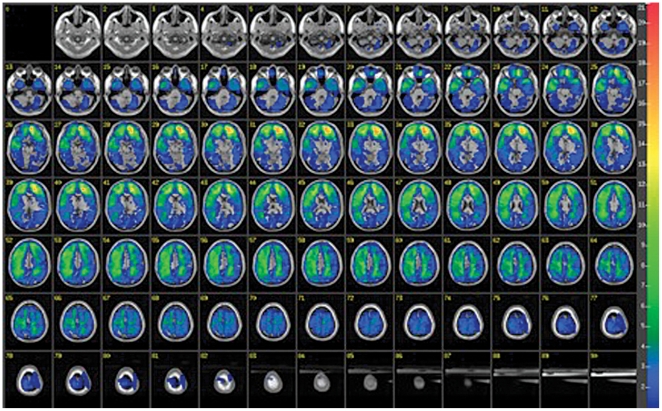
Overlay lesion map for all 109 subjects. Lesion map for the 109 subjects where lesions were overlaid on a standard brain template. The number of overlapping lesions is illustrated by different colors coding increasing frequencies from blue to red.

The overall lesion map of all subjects analyzed by ABLe software is presented in **Figure 4** and shows a primarily frontal distribution. We performed a VSLM analysis to investigate the effect of lesion location on AFQT performance for either PII or PIII. We repeated this analysis with subgroups based on the different genotypes for SNP rs7124442 and SNP rs1519480 and did not find an association with lesion location.

**Table 8:** Frequencies of haplotypes: Only haplotypes with frequencies ≥0.1 were used.

Haplotype*	Freq, Total	Std.Error, Total	Freq, Controls	Std.Error, Control	Freq, Injured	Std.Error, Injured
112122	0.44	0.003	0.49	0.003	0.43	0.003
111222	0.20	0.003	0.19	0.003	0.20	0.003
222212	0.20	0.003	0.20	0.005	0.20	0.003
222211	0.10	0.003	0.10	0.005	0.10	0.003
Block 1 **						
112	0.48	0.0007	0.51	0.0002	0.48	0.0009
222	0.31	0.0007	0.29	0.0002	0.32	0.0009
111	0.21	0.0007	0.19	0.0002	0.21	0.0009
Block 2***						
122	0.45	0.0022	0.50	0.0033	0.44	0.0023
222	0.23	0.0024	0.23	0.0029	0.23	0.0027
212	0.21	0.0019	0.17	0.0041	0.22	0.0018
211	0.10	0.0017	0.10	0.0039	0.10	0.0017

*Frequencies of 6-SNP haplotypes (Fig.1).

**Block 1 Haplotype Frequencies (Fig.1 left panel, SNPs 1–3).

***Block 2 Haplotype Frequencies (Fig.1 right panel, SNPs 4–6).

**Table 9:** Association analysis of haplotype and AFQT z-scores at different time points.

Time of AFQT	Haplotype	Mean±SD AFQT z-scores	N	Statistics	F	P
			***All 6SNPs***			
	111222	0,20±0.89	39			
Pre-Injury	112122	−0.07±0.93	98			
	222211	0,22±0.88	24			
	222212	0,04±0.98	40			
	111222	−1,2328	39	**Time:** *F*(2,197) = 64.2, *P*<0.001*		
PII	112122	−0.07±0.95	98	**Haplotype:** *F*(1,197) = 2.5, *P* = 0.063		
	222211	−0,43±1.26	24	**Time × Haplotype:** *F*(6,197) = 1.2, *P* = 0..319		
	222212	−0,71±1.2	40			
	111222	−0.35±0.91	39			
PIII	112122	−0,80±0.96	98			
	222211	−0,54±1.15	24			
	222212	−0,83±1.07	40			
			* ***Block 1***			
	111	0,30±0.89	36			
Pre-Injury	112	−0,04±0.92	96			
	222	0,16±0.92	64			
	111	−0,09±0.99	36			
PII	112	−0,46±0.98	96	**Time**: *F*(2,193) = 64.8, *P*<0.001*		
	222	−0,59±1.21	64	**Haplotype**: *F*(2,192) = 2.4, *P* = 0.089		
	111	−0,26±0.96	36	**Time × Haplotype**: *F*(2,193) = 2.1, *P* = 0.086		
PIII	112	−0,64±0.92	96			
	222	−0,69±1.09	64			
			** ***Block 2***			
	122	−0.04±0.89	91			
Pre-Injury	211	0,28±0.82	21			
	212	0,07±0.96	43			
	222	0,31±0.95	41			
	122	−0,49±0.96	91	**Time**: *F*(2,192) = 63.4, *P*<0.001*		
PII	211	−0,41±1.31	21	**Haplotype**: *F*(3,192) = 2.2, *P* = 0.094		
	212	−0,64±1.18	43	**Time × Haplotype**: *F*(6,192) = 0.8, *P* = 0.526		
	222	−0,11±1.10	41			
	122	−0,67±0.89	91			
PIII	211	−0,46±1.16	21			
	212	−0,75±1.09	43			
	222	−0,29±0.98	41			
			*** **Block 1 combined**			
Pre-Injury	111	0,06±0.92	132			
	112+222	0,16±0.92	64	**Time**: F(2,194) = 78.7, P<0.001*		
PII	111	−0,36±0.99	132	**Time × Haplotype**: F(2,194) = 4.1, P<0.017*		
	112+222	−0,59±1.21	64	**Haplotype**: F(1,194) = 0.5, P = 0.333		
PIII	111	−0,55±0.94	132			
	112+222	−0,69±1.09	64			

For graphical representation see Fig 3.

* Block 1 includes SNPs rs1519480, rs7124442 and rs6265.

** Block 2 includes SNPs rs7934165, rs11030121 and rs908867.

*** haplotypes 112 and 222 of block 1 were combined and compared with haplotype 111.

* Effect survives Bonferroni correction (P = 0.05/3 ≈ 0.017).

## Discussion

The results indicate that the BDNF polymorphism has an effect on the early-recovery of general cognitive intelligence after penetrating TBI, although it has no influence on pre-injury intelligence or the stability of general intelligence following recovery of function. Prior studies could not find a significant association between the Val66met BDNF genotype and overall intelligence in a healthy population [14], [26]. A previous study from our group did not find a significant association between post-injury intelligence and the Val66met BDNF genotype [5]. However, looking at additional SNPs we provide evidence that rs7124442 and rs1519480 have an effect on recovery of general cognitive intelligence and that this influence is probably a lesion-induced plasticity that is decreased overtime. Our haplotype analysis confirmed this significant association.

As found in prior studies, we confirmed that pre-injury intelligence is the most consistent and vital predictor of cognitive outcome after TBI [4], [5], [38], [39]. The next best predictor at the Phase II evaluation was the BDNF genotype. There were no effects of the ApoE e4 allele or COMT Val158Met on recovery of general cognitive intelligence. The ApoE e4 allele has shown to be one of the strongest genetic predictors of poor outcome following TBI [40], [41], [42]. Several association studies have investigated the role of the ApoE gene polymorphism in subjects with closed TBI but overall the findings are inconclusive [43], [44].

An analysis of the association of lesion location and overall AFQT score did not show any specific location to be significant. Previous studies have shown lesion location to be the least predictive of performance outcome of overall post injury intelligence [3], [4]. Relations have mainly been found between subtest outcomes and specific injured lobe or hemisphere and not full-scale IQ or general intelligence [3], [45], [46]. However in healthy people, studies show full-scale IQ to be significantly correlated with intracranial, cerebral, temporal lobe and hippocampal volume as well as to overall gray matter volume [47], [48], [49]. Several neuroimaging studies on intelligence and reasoning has generated a suggestive model of the Parieto-Frontal Integration Theory (P-FIT) [50]. This model explains the individual differences in intelligence by the interaction between association cortices within parietal and frontal brain regions that are effectively linked by white matter structures. While we did not find a specific lesion location to be significant, the percentage of total volume loss was one of the main predictors of general intelligence at Phases II and III.

Prior studies have shown that aging decreases the brain's volume of gray and white matter and age-related cognitive decline is associated with atrophic changes in the brain [51], [52], [53]. Aging is also associated with decreasing neurotransmitter levels [54], neuronal loss and shrinking neuronal size [55], [56] as well as reduced synaptic density [57]. However there is very little known about BDNF in normal aging brain. A study in monkey showed low levels of BDNF during aging [58]. A recent study in healthy individuals revealed a faster rate of executive control decline on a task-switching task that varied as a function of the val66met BDNF polymorphism in age >65 [59]. IQ measurements using the Wechsler Adult Intelligence Scales (WAIS) have shown that IQ declines with age and that age-related decline is more pronounced on certain subtests of WAIS [60], [61], [62], [63], [64]. In the current study there were no significant differences between the genotypes with respect to degree of atrophy and total brain volume loss. None of the subjects with penetrating head injury had MMSE scores lower than 25. Scores below 25 are associated with mild dementia and age-related cognitive impairment [28]. There was no age difference between the different genotypes.

Furthermore, at the time of the Phase II AFQT testing, the subjects were in their forties, long before the estimated age of 50 at which accelerated cell loss occurs [65], [66]. More importantly the key BDNF genotypes contributed 5% of the variance in recovery of general intelligence even when controlling for age and education. Thus, we do not believe that our findings at Phase II can be explained by age-related cognitive decline. Rather, we speculate that it is indicative of lesion-induced plasticity in which BDNF is known to play a crucial role.

Previous studies have investigated the association of the rs7124442 SNP to eating disorders, cognitive abilities in the elderly, major depression, susceptibility to asthma, Parkinson's disease, autism, Alzheimer's disease and migraine [67], [68], [69], [70], [71], [72], [73]. The only significant associations identified were to eating disorders and high plasma BDNF levels [67] as well as a significant negative influence of TT genotype on treatment response to SSRI, opposed to CC [69]. The BDNF gene contains 11 exons and spans about 70 kb. 9 of these exons are transcription start points, each of which are associated with a functional promoter [74]. The rs7124442 SNP sequence is located in the 3′-UTR region. These sequences are a section of mRNA that follows a coding region and contains several regulatory sequences. 3′-UTR sequences might affect the activity-dependent BDNF mRNA targeting, translation and/or degradation at active synaptic sites and may also influence the proBDNF transcript. The SNP rs1519480 is located in the 3′-flanking region located in highly conserved regions which suggests that this SNP plays an important functional role. The rs1519480 SNP is associated with bipolar disorder [75].

Given that BDNF and its high affinity receptor tropomycin-related kinase B (TrkB) play a crucial role in axon guidance and growth as well as synapse formation and plasticity [76], [77], [78], [79] it is straightforward to think that polymorphisms at this gene would influence recovery of function after a brain injury, particularly in the close period of time when extensive synaptic remodeling might occur. BDNF mRNA expression is mostly limited to neurons and there are only a few brain areas where BDNF mRNA is not detected [80], [81], [82]. Although there are quite limited studies on the role of BDNF and its receptors following TBI, they indicate specific temporal and spatial changes in BDNF expression. Using lateral fluid percussion model in rats, a decrease of BDNF and its receptor TrkB could be detected in the injured area while there was an increase in remote areas hours post-injury [83]. Increased expression of BDNF and TrkB has also been observed in hippocampus hours after injury [84] and interestingly exercise following TBI increased BDNF in CA1 of hippocampus [85]. However we know little about the consequences of these alterations following TBI: are they compensatory responses, what role do they play in cell death and synaptic plasticity and in particular what are the long-term effects? We are investigating these questions in our penetrating rodent TBI model using histopathological, genetic and behavioral studies to get a deeper insight into the role of BDNF following TBI [86].

A century ago, Cajal proposed that the brain possesses the ability to remodel and strengthen neuronal cortical connections based on experience [87], [88], [89] and there are several studies that demonstrate the central role of BDNF in brain plasticity. It regulates vesicle docking in ongoing synaptic functioning and enhances synaptic transmission [90], [91]. Furthermore, BDNF is essential for synaptic remodeling in the adult hippocampus [92].

While injury-induced plasticity may last for a considerable time, it appears reduced with increasing age. One reason may be that proliferation of progenitor cells decreases with age [93], [94], [95] and that BDNF acts in conjunction with this proliferation. In a study on rats subjected to traumatic brain injury at the age of 4 months, a sixfold proliferation of neuroblasts and glia persisted in the subventicular zone 1 year after the trauma [96]. In contrast, noninjured control rats exhibited a decrease of neuroblasts and glia with age. In an additional study, lesion-induced sprouting was seen in the hippocampus of young and adult mice but not observed in aged mice [97].

In conclusion, the results described in this paper indicate that BDNF polymorphism plays a modest but significant role in lesion-induced plasticity, which declines over time. Furthermore, the findings show the impact of two specific SNPs on the recovery of general intelligence. It is possible that polymorphisms in the BDNF gene contribute to functional enhancement or reconstruction of impaired neuronal circuitry or that endogenous neurogenesis is enhanced. Identifying the underlying mechanism of this BDNF effect should provide insight into an important aspect of cognitive recovery after TBI and the value of assessing phenotype risk in TBI subjects.

## References

[1] Faul M XL, Wald MM, Coronado VG (2010). Traumatic brain injury in the United States: emergency department visits, hospitalizations, and deaths.. Centers for Disease Control and Prevention, National Center for Injury Prevention and Control.

[2] Maas AI, Stocchetti N, Bullock R (2008). Moderate and severe traumatic brain injury in adults.. Lancet Neurol.

[3] Grafman J, Jonas BS, Martin A, Salazar AM, Weingartner H (1988). Intellectual function following penetrating head injury in Vietnam veterans.. Brain 111 (Pt.

[4] Grafman J, Salazar A, Weingartner H, Vance S, Amin D (1986). The relationship of brain-tissue loss volume and lesion location to cognitive deficit.. J Neurosci.

[5] Raymont V, Greathouse A, Reding K, Lipsky R, Salazar A (2008). Demographic, structural and genetic predictors of late cognitive decline after penetrating head injury.. Brain.

[6] Strenze T (2007). Intelligence and socioeconomic success: A meta-analytic review of longitudinal research.. Intelligence.

[7] Gottfredson L (1997). Mainstream Science on Intelligence: An Editorial With 52 Signatories, History, and Bibliography.. Intelligence.

[8] Leibrock J, Lottspeich F, Hohn A, Hofer M, Hengerer B (1989). Molecular cloning and expression of brain-derived neurotrophic factor.. Nature.

[9] Lipsky RH, Marini AM (2007). Brain-derived neurotrophic factor in neuronal survival and behavior-related plasticity.. Ann N Y Acad Sci.

[10] Thoenen H (1995). Neurotrophins and neuronal plasticity.. Science.

[11] Poo MM (2001). Neurotrophins as synaptic modulators.. Nat Rev Neurosci.

[12] Huang EJ, Reichardt LF (2001). Neurotrophins: roles in neuronal development and function.. Annu Rev Neurosci.

[13] Okada T, Hashimoto R, Numakawa T, Iijima Y, Kosuga A (2006). A complex polymorphic region in the brain-derived neurotrophic factor (BDNF) gene confers susceptibility to bipolar disorder and affects transcriptional activity.. Mol Psychiatry.

[14] Egan MF, Kojima M, Callicott JH, Goldberg TE, Kolachana BS (2003). The BDNF val66met polymorphism affects activity-dependent secretion of BDNF and human memory and hippocampal function.. Cell.

[15] Hariri AR, Goldberg TE, Mattay VS, Kolachana BS, Callicott JH (2003). Brain-derived neurotrophic factor val66met polymorphism affects human memory-related hippocampal activity and predicts memory performance.. J Neurosci.

[16] Pezawas L, Verchinski BA, Mattay VS, Callicott JH, Kolachana BS (2004). The brain-derived neurotrophic factor val66met polymorphism and variation in human cortical morphology.. J Neurosci.

[17] Szeszko PR, Lipsky R, Mentschel C, Robinson D, Gunduz-Bruce H (2005). Brain-derived neurotrophic factor val66met polymorphism and volume of the hippocampal formation.. Mol Psychiatry.

[18] Bueller JA, Aftab M, Sen S, Gomez-Hassan D, Burmeister M (2006). BDNF Val66Met allele is associated with reduced hippocampal volume in healthy subjects.. Biol Psychiatry.

[19] Huang R, Huang J, Cathcart H, Smith S, Poduslo SE (2007). Genetic variants in brain-derived neurotrophic factor associated with Alzheimer's disease.. J Med Genet.

[20] Hall D, Dhilla A, Charalambous A, Gogos JA, Karayiorgou M (2003). Sequence variants of the brain-derived neurotrophic factor (BDNF) gene are strongly associated with obsessive-compulsive disorder.. Am J Hum Genet.

[21] Ribases M, Gratacos M, Armengol L, de Cid R, Badia A (2003). Met66 in the brain-derived neurotrophic factor (BDNF) precursor is associated with anorexia nervosa restrictive type.. Mol Psychiatry.

[22] Sklar P, Gabriel SB, McInnis MG, Bennett P, Lim YM (2002). Family-based association study of 76 candidate genes in bipolar disorder: BDNF is a potential risk locus. Brain-derived neutrophic factor.. Mol Psychiatry.

[23] Neves-Pereira M, Mundo E, Muglia P, King N, Macciardi F (2002). The brain-derived neurotrophic factor gene confers susceptibility to bipolar disorder: evidence from a family-based association study.. Am J Hum Genet.

[24] Krueger F, Pardini M, Huey ED, Raymont V, Solomon J (2011). The role of the Met66 brain-derived neurotrophic factor allele in the recovery of executive functioning after combat-related traumatic brain injury.. J Neurosci.

[25] Hicks RR, Numan S, Dhillon HS, Prasad MR, Seroogy KB (1997). Alterations in BDNF and NT-3 mRNAs in rat hippocampus after experimental brain trauma.. Brain Res Mol Brain Res.

[26] Tsai SJ, Hong CJ, Yu YW, Chen TJ (2004). Association study of a brain-derived neurotrophic factor (BDNF) Val66Met polymorphism and personality trait and intelligence in healthy young females.. Neuropsychobiology.

[27] Raymont V, Salazar AM, Krueger F, Grafman J (2011). "Studying injured minds" - the Vietnam head injury study and 40 years of brain injury research.. Frontiers in neurology (Neurotrauma).

[28] Folstein MF, Folstein SE, McHugh PR (1975). "Mini-mental state". A practical method for grading the cognitive state of patients for the clinician.. J Psychiatr Res.

[29] Wechsler D (1997). Wechsler Memory Scale - III..

[30] Makale M, Solomon J, Patronas NJ, Danek A, Butman JA (2002). Quantification of brain lesions using interactive automated software.. Behav Res Methods Instrum Comput.

[31] Solomon J, Raymont V, Braun A, Butman JA, Grafman J (2007). User-friendly software for the analysis of brain lesions (ABLe).. Comput Methods Programs Biomed.

[32] Woods RP, Mazziotta JC, Cherry SR (1993). MRI-PET registration with automated algorithm.. J Comput Assist Tomogr.

[33] Tzourio-Mazoyer N, Landeau B, Papathanassiou D, Crivello F, Etard O (2002). Automated anatomical labeling of activations in SPM using a macroscopic anatomical parcellation of the MNI MRI single-subject brain.. Neuroimage.

[34] Hodgkinson CA, Yuan Q, Xu K, Shen PH, Heinz E (2008). Addictions biology: haplotype-based analysis for 130 candidate genes on a single array.. Alcohol Alcohol.

[35] Stephens M, Smith NJ, Donnelly P (2001). A new statistical method for haplotype reconstruction from population data.. Am J Hum Genet.

[36] Gabriel SB, Schaffner SF, Nguyen H, Moore JM, Roy J (2002). The structure of haplotype blocks in the human genome.. Science.

[37] Cohen J (1988). Statistical power analysis for behavioral sciences..

[38] Gao B, Jiang S, Wang X, Chen J (2000). The role of pre-injury IQ in the determination of intellectual impairment from traumatic head injury.. J Neuropsychiatry Clin Neurosci.

[39] Kesler SR, Adams HF, Blasey CM, Bigler ED (2003). Premorbid intellectual functioning, education, and brain size in traumatic brain injury: an investigation of the cognitive reserve hypothesis.. Appl Neuropsychol.

[40] Teasdale GM, Nicoll JA, Murray G, Fiddes M (1997). Association of apolipoprotein E polymorphism with outcome after head injury.. Lancet.

[41] Lichtman SW, Seliger G, Tycko B, Marder K (2000). Apolipoprotein E and functional recovery from brain injury following postacute rehabilitation.. Neurology.

[42] Nathoo N, Chetty R, van Dellen JR, Barnett GH (2003). Genetic vulnerability following traumatic brain injury: the role of apolipoprotein E.. Mol Pathol.

[43] Jordan BD (2007). Genetic influences on outcome following traumatic brain injury.. Neurochem Res.

[44] Dardiotis E, Fountas KN, Dardioti M, Xiromerisiou G, Kapsalaki E (2010). Genetic association studies in patients with traumatic brain injury.. Neurosurg Focus.

[45] Warrington EK, James M, Maciejewski C (1986). The WAIS as a lateralizing and localizing diagnostic instrument: a study of 656 patients with unilateral cerebral lesions.. Neuropsychologia.

[46] Glascher J, Rudrauf D, Colom R, Paul LK, Tranel D (2010). Distributed neural system for general intelligence revealed by lesion mapping.. Proc Natl Acad Sci U S A.

[47] Andreasen NC, Flaum M, Swayze V, 2nd, O'Leary DS, Alliger R (1993). Intelligence and brain structure in normal individuals.. Am J Psychiatry.

[48] Toulopoulou T, Grech A, Morris RG, Schulze K, McDonald C (2004). The relationship between volumetric brain changes and cognitive function: a family study on schizophrenia.. Biol Psychiatry.

[49] Witelson SF, Beresh H, Kigar DL (2006). Intelligence and brain size in 100 postmortem brains: sex, lateralization and age factors.. Brain.

[50] Jung RE, Haier RJ (2007). The Parieto-Frontal Integration Theory (P-FIT) of intelligence: converging neuroimaging evidence.. Behav Brain Sci.

[51] Good CD, Johnsrude IS, Ashburner J, Henson RN, Friston KJ (2001). A voxel-based morphometric study of ageing in 465 normal adult human brains.. Neuroimage.

[52] Resnick SM, Pham DL, Kraut MA, Zonderman AB, Davatzikos C (2003). Longitudinal magnetic resonance imaging studies of older adults: a shrinking brain.. J Neurosci.

[53] Raz N, Rodrigue KM (2006). Differential aging of the brain: patterns, cognitive correlates and modifiers.. Neurosci Biobehav Rev.

[54] Volkow ND, Wang GJ, Fowler JS, Ding YS, Gur RC (1998). Parallel loss of presynaptic and postsynaptic dopamine markers in normal aging.. Ann Neurol.

[55] Flood DG, Coleman PD (1988). Neuron numbers and sizes in aging brain: comparisons of human, monkey, and rodent data.. Neurobiol Aging.

[56] Shimada A (1999). Age-dependent cerebral atrophy and cognitive dysfunction in SAMP10 mice.. Neurobiol Aging.

[57] Terry RD, Katzman R (2001). Life span and synapses: will there be a primary senile dementia?. Neurobiol Aging.

[58] Hayashi M, Mistunaga F, Ohira K, Shimizu K (2001). Changes in BDNF-immunoreactive structures in the hippocampal formation of the aged macaque monkey.. Brain Res.

[59] Erickson KI, Kim JS, Suever BL, Voss MW, Francis BM (2008). Genetic contributions to age-related decline in executive function: a 10-year longitudinal study of COMT and BDNF polymorphisms.. Front Hum Neurosci.

[60] Ronnlund M, Nilsson L (2006). Adult life-span patterns in WAIS-R Block Design performance: Cross-sectional versus longitudinal age gradients and relations to demographic factors.. Intelligence.

[61] Wechsler D (1939). The Measurement of Adult Intelligence..

[62] Wechsler D (1958). The measurement and appraisal of adult intelligence..

[63] Ryan JJ, Sattler JM, Lopez SJ (2000). Age effects on Wechsler Adult Intelligence Scale-III subtests.. Arch Clin Neuropsychol.

[64] Kaufman A, Lichtenberger E (1990). Assessing adolescent and adult intelligence..

[65] DeCarli C, Massaro J, Harvey D, Hald J, Tullberg M (2005). Measures of brain morphology and infarction in the framingham heart study: establishing what is normal.. Neurobiol Aging.

[66] Scahill RI, Frost C, Jenkins R, Whitwell JL, Rossor MN (2003). A longitudinal study of brain volume changes in normal aging using serial registered magnetic resonance imaging.. Arch Neurol.

[67] Mercader JM, Ribases M, Gratacos M, Gonzalez JR, Bayes M (2007). Altered brain-derived neurotrophic factor blood levels and gene variability are associated with anorexia and bulimia.. Genes Brain Behav.

[68] Miyajima F, Ollier W, Mayes A, Jackson A, Thacker N (2008). Brain-derived neurotrophic factor polymorphism Val66Met influences cognitive abilities in the elderly.. Genes Brain Behav.

[69] Domschke K, Lawford B, Laje G, Berger K, Young R (2010). Brain-derived neurotrophic factor ( BDNF) gene: no major impact on antidepressant treatment response.. Int J Neuropsychopharmacol.

[70] Szczepankiewicz A, Rose-Zerilli MJ, Barton SJ, Holgate ST, Holloway JW (2009). Association analysis of brain-derived neurotrophic factor gene polymorphisms in asthmatic families.. Int Arch Allergy Immunol.

[71] Xiromerisiou G, Hadjigeorgiou GM, Eerola J, Fernandez HH, Tsimourtou V (2007). BDNF tagging polymorphisms and haplotype analysis in sporadic Parkinson's disease in diverse ethnic groups.. Neurosci Lett.

[72] Nishimura K, Nakamura K, Anitha A, Yamada K, Tsujii M (2007). Genetic analyses of the brain-derived neurotrophic factor (BDNF) gene in autism.. Biochem Biophys Res Commun.

[73] Lemos C, Mendonca D, Pereira-Monteiro J, Barros J, Sequeiros J (2010). BDNF and CGRP interaction: implications in migraine susceptibility.. Cephalalgia.

[74] Pruunsild P, Kazantseva A, Aid T, Palm K, Timmusk T (2007). Dissecting the human BDNF locus: bidirectional transcription, complex splicing, and multiple promoters.. Genomics.

[75] Liu L, Foroud T, Xuei X, Berrettini W, Byerley W (2008). Evidence of association between brain-derived neurotrophic factor gene and bipolar disorder.. Psychiatr Genet.

[76] Frisen J, Verge VM, Cullheim S, Persson H, Fried K (1992). Increased levels of trkB mRNA and trkB protein-like immunoreactivity in the injured rat and cat spinal cord.. Proc Natl Acad Sci U S A.

[77] Huang EJ, Reichardt LF (2003). Trk receptors: roles in neuronal signal transduction.. Annu Rev Biochem.

[78] Luikart BW, Parada LF (2006). Receptor tyrosine kinase B-mediated excitatory synaptogenesis.. Prog Brain Res.

[79] Lu B (2003). Pro-region of neurotrophins: role in synaptic modulation.. Neuron.

[80] Katoh-Semba R, Takeuchi IK, Semba R, Kato K (1997). Distribution of brain-derived neurotrophic factor in rats and its changes with development in the brain.. J Neurochem.

[81] Conner JM, Lauterborn JC, Yan Q, Gall CM, Varon S (1997). Distribution of brain-derived neurotrophic factor (BDNF) protein and mRNA in the normal adult rat CNS: evidence for anterograde axonal transport.. J Neurosci.

[82] Ernfors P, Ibanez CF, Ebendal T, Olson L, Persson H (1990). Molecular cloning and neurotrophic activities of a protein with structural similarities to nerve growth factor: developmental and topographical expression in the brain.. Proc Natl Acad Sci U S A.

[83] Hicks RR, Li C, Zhang L, Dhillon HS, Prasad MR (1999). Alterations in BDNF and trkB mRNA levels in the cerebral cortex following experimental brain trauma in rats.. J Neurotrauma.

[84] Hicks RR, Martin VB, Zhang L, Seroogy KB (1999). Mild experimental brain injury differentially alters the expression of neurotrophin and neurotrophin receptor mRNAs in the hippocampus.. Exp Neurol.

[85] Hicks RR, Boggs A, Leider D, Kraemer P, Brown R (1998). Effects of Exercise Following Lateral Fluid Percussion Brain Injury in Rats.. Restor Neurol Neurosci.

[86] Risling M, Plantman S, Angeria M, Rostami E, Bellander BM (2011). Mechanisms of blast induced brain injuries, experimental studies in rats.. Neuroimage.

[87] Pascual-Leone A, Amedi A, Fregni F, Merabet LB (2005). The plastic human brain cortex.. Annu Rev Neurosci.

[88] Will B, Galani R, Kelche C, Rosenzweig MR (2004). Recovery from brain injury in animals: relative efficacy of environmental enrichment, physical exercise or formal training (1990-2002).. Prog Neurobiol.

[89] Seitz RJ, Kleiser R, Butefisch CM (2005). Reorganization of cerebral circuits in human brain lesion.. Acta.

[90] Pozzo-Miller LD, Gottschalk W, Zhang L, McDermott K, Du J (1999). Impairments in high-frequency transmission, synaptic vesicle docking, and synaptic protein distribution in the hippocampus of BDNF knockout mice.. J Neurosci.

[91] Tyler WJ, Pozzo-Miller LD (2001). BDNF enhances quantal neurotransmitter release and increases the number of docked vesicles at the active zones of hippocampal excitatory synapses.. J Neurosci.

[92] Heldt SA, Stanek L, Chhatwal JP, Ressler KJ (2007). Hippocampus-specific deletion of BDNF in adult mice impairs spatial memory and extinction of aversive memories.. Mol Psychiatry.

[93] Kolb B (1995). Brain plasticity and behavior..

[94] Luebke JI, Rosene DL (2003). Aging alters dendritic morphology, input resistance, and inhibitory signaling in dentate granule cells of the rhesus monkey.. J Comp Neurol.

[95] Kuhn HG, Dickinson-Anson H, Gage FH (1996). Neurogenesis in the dentate gyrus of the adult rat: age-related decrease of neuronal progenitor proliferation.. J Neurosci.

[96] Chen XH, Iwata A, Nonaka M, Browne KD, Smith DH (2003). Neurogenesis and glial proliferation persist for at least one year in the subventricular zone following brain trauma in rats.. J Neurotrauma.

[97] Kadish I, van Groen T (2009). Lesion-induced hippocampal plasticity in transgenic Alzheimer's disease mouse models: influences of age, genotype, and estrogen.. J Alzheimers Dis.

